# Analysis of the Specific Expression Profile of Immune Cells in Infants and Young Children Infected with RSV and Construction of a Disease Prediction Model

**DOI:** 10.3390/tropicalmed11010010

**Published:** 2025-12-29

**Authors:** Kai Ren, Honggang Sun, Tian Ren, Kailun Ma, Jizheng Chen

**Affiliations:** 1 State Key Laboratory of Respiratory Disease, National Clinical Research Center for Respiratory Disease, Guangzhou Institute of Respiratory Health, the First Affiliated Hospital of Guangzhou Medical University, Guangzhou 510005, China; 15179183697@163.com (K.R.); ren_tian@gzlab.ac.cn (T.R.); 2 Guangzhou National Laboratory, No. 9 Xing Dao Huan Bei Road, Guangzhou International Bio Island, Guangzhou 510005, China; higsir@126.com (H.S.); ma_kailun@gzlab.ac.cn (K.M.); 3 State Key Laboratory of Virology, Wuhan Institute of Virology, Center for Biosafety Mega-Science, Chinese Academy of Sciences, Wuhan 430071, China

**Keywords:** RSV, ALRI, Th1/Th2, immune cell landscape, WGCNA, machine learning algorithm models

## Abstract

It has been demonstrated that infants and young children exhibit immune tolerance as a consequence of immature immune systems, which are characterized by a natural Th2 bias. RSV infection has been reported to result in acute lower respiratory infection (ALRI), while formalin-inactivated vaccination has been observed to exacerbate Th2 responses, consequently leading to enhanced respiratory disease (ERD). Transcriptomic data from three independent cohorts of RSV-infected infants were analyzed (GSE246622 served as the discovery and train set; GSE105450 and GSE188427 were used as validation sets). Immune infiltration analysis revealed immunological characteristics, which were then used to perform unsupervised clustering using feature-related genes. WGCNA was used to identify co-expressed gene modules, while Mfuzz and TCseq were employed to analyze temporal expression patterns. Machine learning models were developed using a refined panel of candidate genes. Severe symptoms of RSV infection exhibited a strong correlation with age, with younger infants demonstrating more intense inflammatory responses from neutrophils, macrophages, mast cells and dendritic cells. A predictive model was constructed using ten co-expressed genes: The following genes were identified: MCEMP1, FCGR1B, ANXA3, FAM20A, CYSTM1, GYG1, ARG1, SLPI, BMX and SMPDL3A. It was observed that infants of a younger demographic demonstrated a heightened degree of immunosuppression and pronounced innate immune activation in patients of severe symptoms with RSV infection. However, eosinophils exhibited minimal involvement in these processes. These gene models pertaining to the neutrophil, macrophage or mast cell was found to be a relatively effective predictor in patients of severe symptoms.

## 1. Introduction

Respiratory syncytial virus (RSV) infection poses a significant threat to the health of infants and young children, which imposes a substantial global burden, with an estimated 33 million new cases of acute lower respiratory infection (ALRI) occurring each year among children under five [1,2]. RSV The clinical presentation typically involves the acute onset of coughing or dyspnea, often accompanied by tachypnea [1,3]. The incidence of severe disease peaks between 2 and 4 months of age, with the majority of critical cases occurring in infants under 6 months [4]. The consequences of this phenomenon are significant, with the number of hospital admissions and mortalities reaching approximately 3.2 million and 120,000 per annum, respectively [1].

Incomplete maturation of the immune system in early life predisposes infants to pulmonary infections by causing a dysfunctional adaptive immune response that fails to generate effective and durable memory [4]. Neonatal immunity is marked by a Th2-polarized predisposition, evident in the cytokine response of TLR-stimulated dendritic cells and monocytes, compounded by a lymphocyte profile rich in recent thymic emigrants that are inherently biased toward Th2 effector differentiation [5,6].

It has been demonstrated that infants’ innate Th2-polarized immunity can result in severe ERD following immunization with early formalin-inactivated RSV vaccines [7]. This is characterized by parenchymal tissue damage, bronchopneumonia with atelectasis/pneumothorax, and pulmonary neutrophilia with macrophage/lymphocyte infiltration and eosinophilia [7]. In addition, the immunological characteristic of infants and young children infected with respiratory syncytial virus (RSV) lies in the incomplete maturation of both innate and adaptive immune systems, which not only results in their relatively weak viral clearance ability but also is often accompanied by excessive inflammatory responses [8]. Upon infection with respiratory syncytial virus (RSV), neonates exhibit reduced Toll-like receptor (TLR) signaling, altered antigen-presenting cell (APC) function, decreased expression of innate antiviral cytokines (interferons), and increased production of inflammation-related factors. This may skew the adaptive immune response toward Th2 and Th17 subsets, impairing the protective antiviral functions of Th1 cells and cytotoxic T lymphocytes (CTLs) [9]. However, the majority of research on RSV infection focuses exclusively on the immunological distinctions between infants and young children, adults, and the elderly, with limited attention paid to the variations among infants of different months within one year of age. Exploration of the immunological characteristics of RSV infection in infants is therefore of paramount importance for the prevention and treatment of antivirals, as well as for the research and development of vaccines in this age stage.

**Figure 1:**
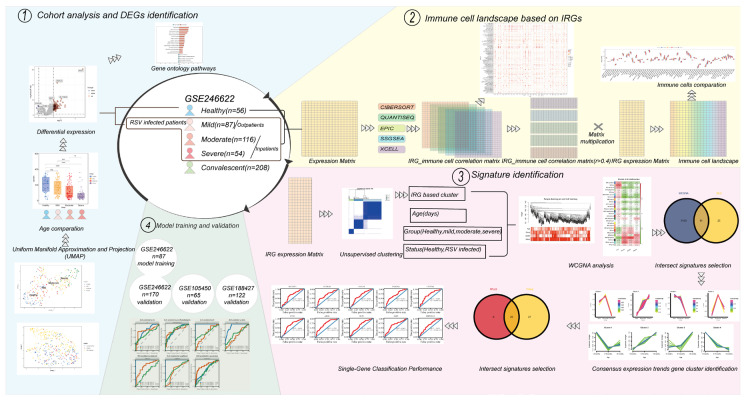
Flow chart for the study (ns > 0.05, ** *p* < 0.01, *** *p* < 0.001, **** *p* < 0.0001; *t*-test).

In this study, we analyzed transcriptome sequencing data from whole blood samples of infants under one year old hospitalized due to RSV infection, which were obtained from an open database GSE246622. Seven machine learning algorithm models were constructed by training on 87 samples from the GSE246622 dataset, with three independent validation sets utilized, including 170 samples from GSE246622, 65 samples from GSE105450, and 122 samples from GSE188427. To clarify the immunological features of infants aged less than one year in the context of RSV infection and develop a predictive model for predicting severe symptoms of RSV infection, the following analytical methods were adopted: immune infiltration analysis, consensus matrix analysis, WGCNA, Mfuzz, TCseq, and machine learning algorithms. We revealed distinct immune cell profiles in infants featuring T/B cell dysfunction with compensatory inflammatory hyperactivation. A total of ten genes, including MCEMP1, FCGR1B, ANXA3, FAM20A, CYSTM1, GYG1, ARG1, SLPI, BMX and SMPDL3A, were found to demonstrate significant correlations with age and severe symptoms of RSV infection, thus providing novel insights with regard to clinical diagnostics, therapeutics and vaccine design. The specific analysis process is shown in Figure 1.

## 2. Materials and Methods

### 2.1. Data Collection and Immune Cell-Related Genes (IRGs)

The RNA expression matrix and the corresponding clinical information were obtained from the public database, GEO, which is maintained by the National Center for Biotechnology Information (NCBI) (https://www.ncbi.nlm.nih.gov/ (accessed on 15 August 2025)). Our study included three transcriptome datasets of RSV-infected infants (GSE246622 [10], GSE188427, GSE105450 [11]). The 47 immune-related genes (IRGs) were derived from a previously published study, where they were utilized as markers for flow cytometry to identify immune cell populations in RSV-infected infants’ blood samples [10].

### 2.2. Analysis of RSV-Infected Cohorts

A total of 533 infants under 12 months from GSE246622 were included in the study, consisting of 56 healthy individuals, 208 convalescent patients, and 257 RSV-infected individuals. The severity of infection was then subjected to further grouping according to ReSVinet score [12], resulting in the identification of 87 mild cases, 116 moderate cases, and 54 severe cases. Principal Component Analysis (PCA) was performed to visualize the sample distribution in each group. The identification of differentially expressed genes (DEGs) between healthy individuals and RSV-infected patients was undertaken in accordance with the following criteria: The first criterion is that |logFC| must be greater than 1; the second is that *p*-value must be less than 0.05. The application of these criteria resulted in the identification of 86 DEGs. The R package 1.28.4 “enrichplot” was utilized to conduct Gene Ontology (GO) enrichment analysis of DEGs. The generation of heatmaps of the DEGs was achieved by utilizing the complexheatmap R package [13].

### 2.3. Immune Cell Landscape

CIBERSORT [14], EPIC [15], QUANTISEQ [16], XCELL [17], and Single Sample Gene Set Enrichment Analysis (ssGSEA) was employed for immune cell infiltration analysis. Following the establishment of correlations between IRGs and various immune cells, a subset exhibiting a correlation coefficient greater than 0.4 was selected. This subset was then utilized for matrix multiplication with the IRG expression matrix to obtain the final results for the immune cell landscape.

### 2.4. Clustering

The R package ‘ConsensusClusterPlus’ [18] was utilized to analyze the GSE246622 cohort, which was divided into three clusters based on the 47 IRGs. The clustering analysis was configured with a maximum of 9 clusters. The process involved 50 iterations of resampling, wherein 80% of the samples were randomly selected in each iteration. Feature sampling was disabled (feature ratio = 1), and the Partitioning Around Medoids (PAM) algorithm was employed using Euclidean distance as the metric. The identification of differentially expressed genes between each cluster was conducted in accordance with the criteria delineated in Section 2.2.

### 2.5. Weighted Correlation Network Analysis (WGCNA) [19]

The R package WGCNA was utilized to investigate the co-expression gene network. The ‘pickSoftThreshold’ function was applied in order to calculate the soft threshold, with the optimal threshold identified as 12. The minimum number of genes that could be accommodated within each module was set at ten. A total of 1161 genes across the five modules were selected for further analysis. Subsequently, 61 genes were selected for further analysis, as they were present in both 86 differentially expressed genes (DEGs) and 1161 genes from the five modules.

### 2.6. Signatures Identification

The R package “Time course sequencing data analysis” (Tcseq) was employed for the purpose of conducting a clustering analysis of the 61 genes that have been demonstrated to be correlated with clinical symptoms. In Tcseq, fuzzy c-means clustering was performed, and the algorithm was configured to generate four gene clusters. In addition, Mfuzz [20] was utilized for the identification of genes whose expression varies with age. The optimal fuzzifier value was determined using the mestimate function, and the number of clusters was set to generate four clusters as output. Four gene clusters exhibited consistent up-regulation or down-regulation, with either worsening symptoms or increasing age identified upon analysis. A total of 33 genes were identified using Tcseq, whereas 52 genes were identified using Mfuzz. Twenty-five candidate signatures exhibiting a continuous trend in both age and clinical symptoms were selected for further analysis.

### 2.7. Receiver Operating Characteristic (ROC) and Predictive Model Construction

The patients were divided into two individuals, namely inpatients and outpatients. The Area Under the Curve (AUC) of the ROC curves was utilized to evaluate the accuracy of the 25 signatures. Signatures with an AUC > 0.65 were selected for the construction of a predictive model. The model was trained on 87 samples from the GSE246622 dataset using 5-fold cross-validation with ten repeats. A total of three independent validation sets were used: 170 samples from GSE246622, 65 samples from GSE105450, and 122 samples from GSE188427. For model construction, we employed and tuned seven distinct machine learning algorithms using the R Mime1 package [21]. The hyperparameter search space for each algorithm was configured as follows. The Naïve Bayes (nb) model was tuned with a Laplace correction (fL) tested at 0, 0.5, 1, 1.5, and 2, while kernel density estimation was enabled with a bandwidth adjustment factor (adjust) ranging from 0.5 to 1.5. For the weighted Support Vector Machine with a radial basis function kernel (svmRadialWeights), we evaluated a sigma parameter from 5 × 10^−4^ to 0.05, a cost parameter from 1 to 20, and class Weights from 0.1 to 10. The Random Forest (rf) algorithm was optimized by varying the number of features considered at each split (mtry), which was sampled across ten values from 2 to 369. The Kernel k-Nearest Neighbors (kknn) model was trained with a fixed distance of 2 and an “optimal” kernel, while the maximum number of neighbors (kmax) was tested with odd values from 5 to 13. Two boosting algorithms were also implemented: AdaBoost, for which the number of iterations (nIter) was tested from 50 to 250 and the method was set to either “Adaboost.M1” or “Real adaboost”; and LogitBoost, which was tuned over a range of nIter values from 11 to 101. Finally, the cancerclass method was applied using the welch.test for feature selection prior to classification.

## 3. Results

### 3.1. Gene Expression Was Different Among Individuals with Different Infection Status and Severity

**Figure 2:**
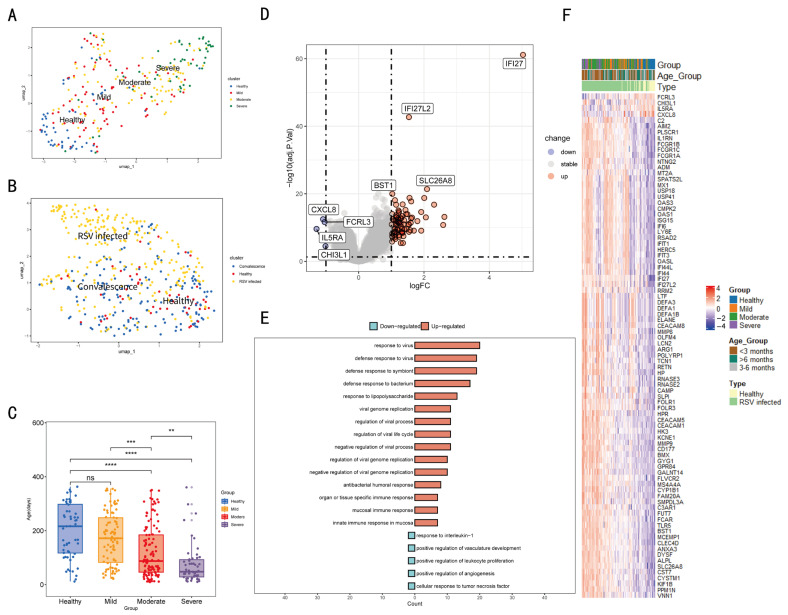
Analysis of differentially expressed genes in the RSV infection transcriptome data. In order to ascertain the differences in mRNA expression levels among healthy (blue dots), mild (orange dots), moderate (red dots), and severe (purple dots) groups, a principal component analysis (PCA) was conducted (**A**). Furthermore, a PCA was conducted in order to ascertain the differences in RSV-infected (red dots), healthy (orange dots), and convalescent (blue dots) statuses (**B**). The correlation between severe symptoms of RSV infection and age is demonstrated in (**C**). As illustrated in (**D**,**F**), the DEGs are displayed on the volcano plot and the heatmap, respectively. The DEGs are analyzed using GO enrichment (**E**). (ns > 0.05, ** *p* < 0.01, *** *p* < 0.001, **** *p* < 0.0001; *t*-test).

The population was divided into three statuses based on infection status: healthy, convalescent, and RSV-infected. The severity of infection in RSV-infected individuals was also divided into three subgroups based on ReSVinet score: mild, moderate, and severe. PCA is performed on the expression profile of different subsets. As demonstrated in Figure 2A, patients with different infection severities were shown to exhibit grouping characteristics, as indicated by PCA. Furthermore, the PCA indicates that RSV-infected individuals are clustered separately from healthy and convalescent individuals (Figure 2B). A significant age difference was found among groups of differing symptom severity, with greater severity associated with younger age (Figure 2C). In order to characterize the transcriptome features of individuals infected with RSV, a screening of 86 differentially expressed genes (|logFC| > 1 & *p* < 0.05) was conducted (Figure 2D,F) Among which, 4 genes (FCRL3, CHI3L1, CXCL8, IL5RA) were downregulated upon RSV infection, while the other 82 differentially expressed genes were all upregulated. GO analysis indicated that the differentially expressed genes between healthy and RSV-infected statuses primarily involved virus-related response genes, which are associated with respiratory mucosal immunity (Figure 2E). This indicates that mucosal immunity plays a core antiviral role in RSV-infected process, and these 86 differentially expressed genes (DEGs) can well reflect the immunological characteristics of infants and young children with RSV infection.

### 3.2. Immune Cell-Related Genes Exhibit the Close Correlation Between the Severity of Symptoms in RSV-Infected Individuals and Immune Cells

**Figure 3:**
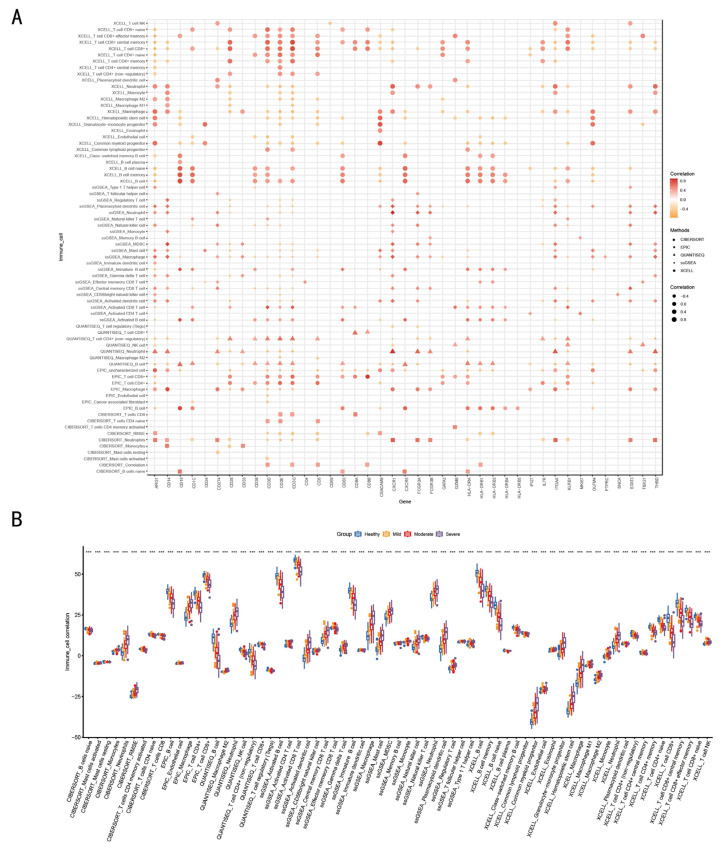
Using 47 immune-related genes to establish the correlation between immune cells and infected populations. Two statuses (healthy and infected individuals) were included, and immune cell infiltration analysis was performed using five methods (CIBERSORT, EPIC, QUANTSEQ, ssGSEA, and xCELL) to obtain the correlation between each sample and various immune cell types. Subsequently, the correlation between immune cell-related genes and various immune cell types was extracted from the aforementioned results. (**A**) was employed to demonstrate the correlation between 41 immune-related factors and immune cells. By multiplying the previously obtained correlation matrix between immune cell-related genes and immune cell types with the expression matrix (which only includes 47 immune cell-related genes) via matrix multiplication, the correlation between each sample and various immune cell types was calculated. Finally, based on the group classification (healthy, mild, moderate, and severe individuals), the differences in immune cell correlations among different subsets were analyzed. (**B**) was used to demonstrate the correlation between healthy (blue column), mild (orange column), moderate (red column), and severe (purple column) populations and immune cells. (*** *p* < 0.001; *t*-test).

Correlations between 47 immune cell-related genes and multiple immune cells were analyzed using five methods: CIBERSORT, EPIC, QUANTISEQ, SSGSEA, and XCELL (Supplementary-Figure S1). Among these, the correlations of 41 genes were greater than 0.4 (Figure 3A). As illustrated in Figure 3B, a correlation was identified between distinct severity groups and immune cells. The functional activity of T and B cells showed a higher correlation with healthy individuals, while the function of neutrophils, macrophages, mast cells, and dendritic cells was more closely associated with severe infections. Because the majority of T and B cell-related functions appeared to be impaired, the functions of neutrophils, macrophages, mast cells, and dendritic cells were enhanced (Figure 3B). In addition, A central finding of this study is the systematic explanation of how the spectrum of immune cells is related to the severity of symptoms in RSV infection. (Figure 3B). The study also reveals that disease severity in immunologically immature infants with RSV infection is associated with two key elements: the spectrum of T and B cells and various types of immunoregulatory cells (Tregs, pDCs, MDSCs, and M2 cells).

### 3.3. Clustering and Annotation of Immune Cell-Related Gene Expression

**Figure 4:**
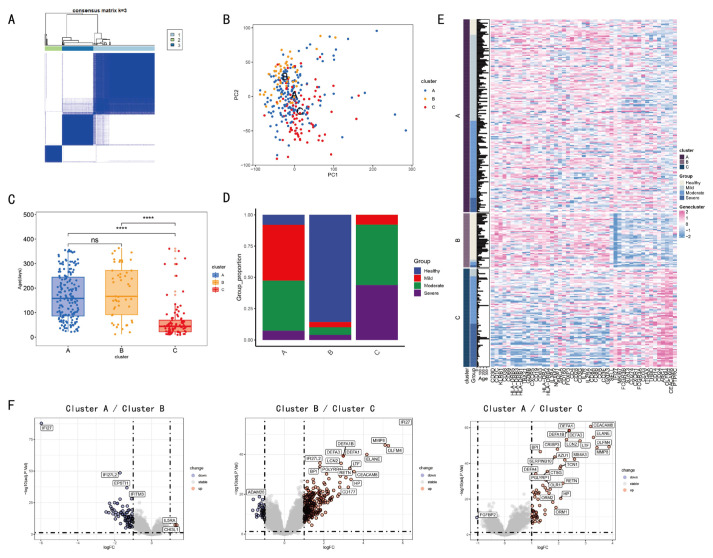
IRGs based clustering and analysis among different clusters. The Consensus matrix analysis categorizes the population into three clusters 1 (light blue), 2 (green), and 3 (blue). (**A**). The following comparison is made among the three clusters by means of PCA. The three clusters, A, B, and C, are represented by different colors in terms of health (blue), mild (red), moderate (green), and severe (purple) (**B**). The correlation between severe symptoms of RSV infection and age is demonstrated in (**C**,**D**). The expression levels of immune cell-related genes are presented using a heat map (**E**). The volcano plot (**F**) demonstrates the differential expression of genes. (ns > 0.05, **** *p* < 0.0001; *t*-test).

The expression matrix of 47 immune cell-related genes was extracted from healthy and infected individuals. Three clusters (1 = B, 2 = C, 3 = A) were identified via consensus matrix and principal component analyses. (Figure 4A,B). Figure 4D illustrates distinct severity of infection (healthy, mild, moderate, severe) in clusters A, B, and C. The subjects in Cluster C, who were of a younger age, exhibited a higher prevalence of severe cases during RSV infection. Furthermore, these subjects showed significant disparities in the expression of 47 IRGs (Figure 4C,E). Cluster B consists mainly of healthy individuals, while Cluster C is predominantly composed of moderate-to-severe infected patients. As shown in Heatmap, the 47 immune-related genes exhibit significant differences in expression levels. In comparison with Cluster B, 30 immune-related genes displayed reduced expression, while another 17 had elevated expression. Moreover, a comparison of each pair of clusters A, B, and C revealed significant disparities in gene expression (|logFC| > 1 and *p* < 0.05) (Figure 4F). A comprehensive compendium of data pertaining to the enrichment of differentially expressed genes in GO analysis is appended (Supplementary-Figure S2).

### 3.4. Co-Expressed Genes Related to Age, Group (Severity of Infection), Cluster, and Status (Infection Status)

**Figure 5:**
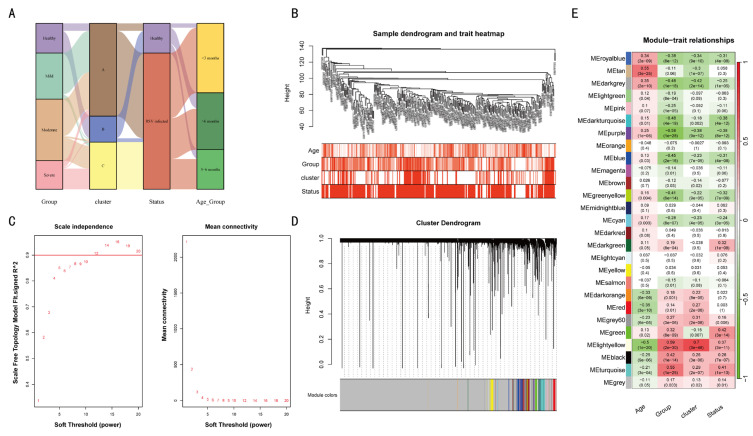
A gene co-expression network was constructed among different classified individuals. The sample grouping and clustering is presented in (**A**,**B**). The mean connectivity and scale-free fit index of soft threshold power are illustrated in (**C**). The hierarchical clustering tree of genes based on topological overlap is confirmed in (**D**). The correlation between these gene modules and age, group, cluster, and status (**E**) is examined.

The WGCNA method was employed to construct a gene co-expression network. For all the previously referenced grouped samples, the Sankey diagram presents the correlations between the four categories: group, cluster, status, and age group (Figure 5A). The severity of infection (group) exhibited a close correlation with age and cluster (Figure 4B). Cluster C had predominantly moderate/severe infections, with most cases in those less than 3 months of age (Figure 5B). Subsequently, we employed dynamic hybridization cutting to construct a hierarchical clustering tree and form gene modules. These branches exhibited numerous genes that demonstrated analogous expression profiles. Each individual gene may be regarded as analogous to a leaf in a tree (Figure 5D). The construction of twenty-seven modules was facilitated with transcriptome data (Figure 5E). “Age” showed a significant negative correlation with the “Group” and “Cluster” in co-expressed genes, indicating their expression levels reflect age and infection severity.

### 3.5. TCseq and Mfuzz Were Used to Analyze the Co-Expressed Gene Module

**Figure 6:**
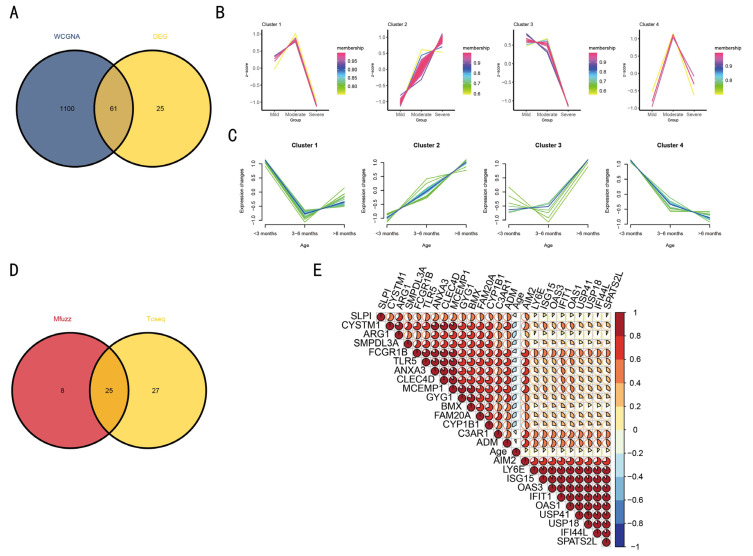
Mfuzz and TCseq were used to analyze differentially co-expressed genes and screen for temporally consistent genes. The Venn diagram analysis of WGCNA and DEG is presented in (**A**). The relationship between age, severity, and gene expression was established by processing the genes using Mfuzz and TCseq (**B**,**C**). The Venn diagram illustrates the linear relationship between the 25 genes and both age and severity (**D**), with the detailed correlation depicted in (**E**).

Among the 1161 genes analyzed using WGCNA, 61 exhibited significant differential expression (Figure 6A). The 61 genes were analyzed by TCseq and Mfuzz, respectively (Figure 6B,C). Both TCseq and Mfuzz are analytical tools based on time-series transcriptome data. By analyzing severe symptoms of RSV infection and age as temporal correlations, it was found that only Cluster 2 identified by both methods exhibited a highly positive correlation. The 25 genes constituting Cluster 2 were found to be contingent on age and the severe symptoms of RSV infection (Figure 6B–E).

### 3.6. Ten Genes That Could Distinguish the Severity of RSV Infection Were Screened

**Figure 7:**
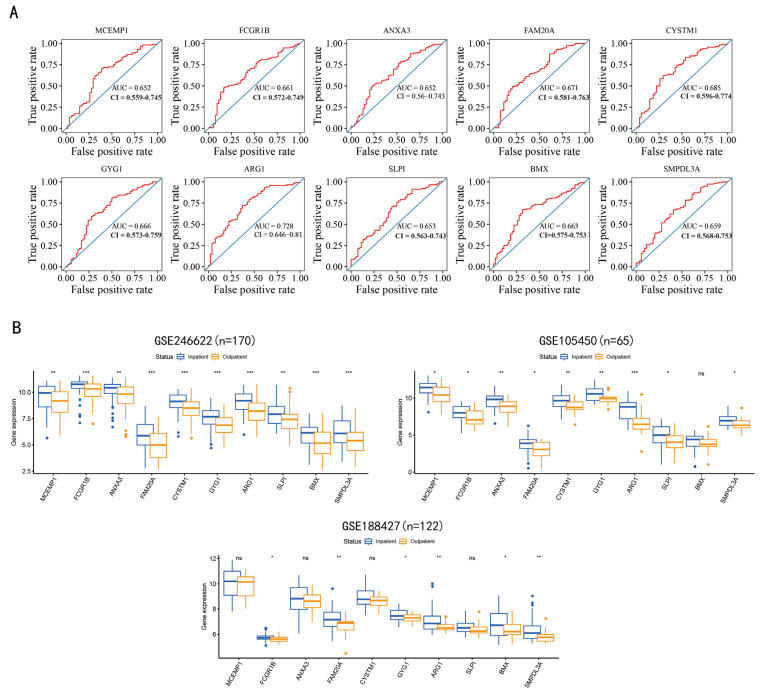
Gene screening for the prediction of RSV infection severity. Inpatients and outpatients were used as positive and negative samples, respectively, and 25 time-series-related co-expressed genes were analyzed, resulting in the identification of 10 genes. The Area Under the Curve (AUC) of the ten genes evaluated by the Receiver Operating Characteristic (ROC) curve is displayed in (**A**). Gene expression levels have been shown to distinguish between discharged and hospitalized patients (**B**), where three whole-blood transcriptome datasets of infants under one year old are involved: GSE246622, GSE105450, GSE188427. (ns > 0.05, * *p* < 0.05, ** *p* < 0.01, *** *p* < 0.001; *t*-test).

The 25 genes that were screened based on Mfuzz and TCseq were utilized for the calculation of the ROC curve. The 10 genes, including MCEMP1, FCGR1B, ANXA3, FAM20A, CYSTM1, GYG1, ARG1, SLPI, BMX and SMPDL3A, which showed an AUC area greater than 0.65, are displayed in Figure 7A. Due to the single-center nature of the analyzed dataset and the limited sample size, the AUC value was relatively low (AUC < 0.7). The expression levels of the ten selected genes in three datasets are illustrated in Figure 7B. Data demonstrate that the differences in the expression levels of the ten genes can distinguish the severity of infection to a certain extent, thereby facilitating the determination of the necessity of hospitalization.

### 3.7. Constructing Machine Learning Algorithm Models

**Figure 8:**
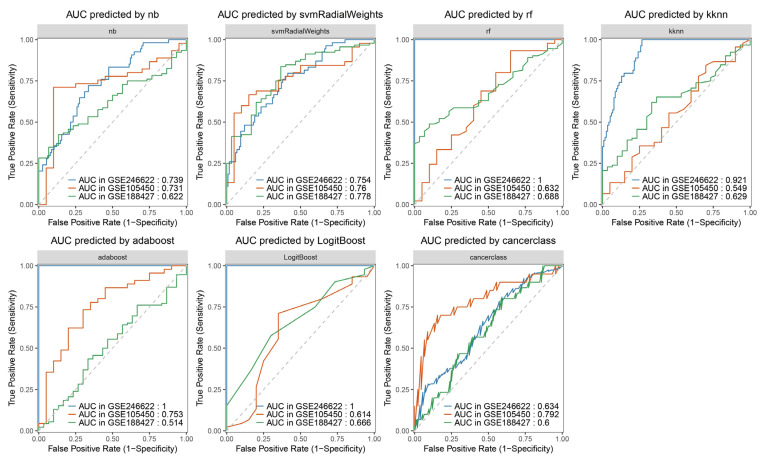
Machine learning algorithm models. ‘nb’: Naive Bayes algorithm is a probabilistic classification algorithm based on Bayes’ theorem and the “conditional independence assumption of features”. ‘svmRadialWeights’: Support Vector Machine (SVM) is capable of processing nonlinear data via kernel functions (Radial Basis Function (RBF) kernel and linear kernel), mapping low-dimensional non-separable data into a high-dimensional space. ‘rf’: Random Forest is a tree-based algorithm based on “ensemble learning”, constructed by multiple decision trees via “bootstrap sampling” and “random feature selection”. ‘kknn’: K-nearest Neighbors is a type of lazy learning algorithm without an explicit training process, with its core being “nearest neighbor voting”. ‘adaboost’: AdaBoost Classification Trees iteratively trains multiple weak classifiers (usually decision stumps) and assigns higher weights to the misclassified samples from the previous iteration. ‘LogitBoost’: Boosted Logistic Regressions is a linear classification algorithm based on the sigmoid function, which outputs classification results in the form of probabilities (usually using 0.5 as the threshold for binary classification). ‘cancerclass’: Cancerclass is constructed based on an ensemble algorithm integrating random forest and support vector machine, which is capable of analyzing gene expression profiles. The dashed line represents a 50% probability for both true and false predictions.

To further improve the credibility of the prediction, the ten screened genes mentioned above were used to construct a classification model for predicting severe symptoms of RSV infection using machine learning algorithms (Figure 8). Consequently, seven machine learning algorithm models were obtained. A portion of the data from the GSE246622 dataset was used as the training set, while the other portion, along with GSE105450 and GSE188427, was employed as the validation set. Compared with the prediction based on the expression level of a single gene, GSE246622, as the validation set, showed that the predictive model constructed using the ten genes together—except for the “cancerclass” model—significantly improved the prediction credibility. The seven constructed predictive models which aimed to determine whether the expression levels of the ten genes can predict severe symptoms of RSV infection in infants under one year old following RSV infection, thereby assisting clinicians in judging the necessity of hospitalization. Integrating data from the seven predictive models can offset the shortcomings of insufficiently significant predictive performance in some models, thereby facilitating more accurate judgment-making. Due to variations in multiple factors—including sample genetic backgrounds, sampling time points, and diagnostic criteria for severe symptoms of RSV infection—this led to suboptimal prediction results with GSE105450 and GSE188427, which also constitutes a limitation to the promotion of these prediction models.

## 4. Discussion

RSV infection in infants and young children has been shown to result in viral lower respiratory tract infections (LRI) characterized by a Th2 tendency and increased pulmonary eosinophils in the immune system [4,22]. This has been demonstrated to result in a significant increase in the rates of hospitalization and mortality among infants and young children [4,22]. Furthermore, the severe ERD effect that has been observed in infants and young children following vaccination with formalin-inactivated vaccine (FI-RSV) has become a significant impediment to the development of RSV vaccines for this demographic, with the result that there are currently no RSV vaccines available on the market [23]. Consequently, it is imperative to undertake comprehensive and meticulous investigation of the immunological characteristics exhibited by infants and young children.

In this study, the objective was to utilize the GSE246622 transcriptome data for the purpose of conducting an expression difference analysis and a GO analysis. This analysis revealed significant disparities in differentially expressed genes among the healthy, convalescent, and RSV-infected status. Furthermore, genes relating to respiratory mucosal immunity were shown to be activated in antiviral immunity-related responses. Cell infiltration analysis indicated that younger infants were more likely to develop severe illness when infected with RSV. As demonstrated in the relevant literature, the immune system of an infant exhibits an immune tolerance state, which in turn results in the suppression of adaptive immunity [4,24,25]. In addition, immune tolerance-related cells, including Tregs, Bregs, and M2 macrophages, have been shown to play an important role in limiting severe allergic reactions and asthma caused by RSV infection [24,26]. The application of correlation analysis revealed a negative correlation between the severity of RSV infection and both T and B cells. Furthermore, immune tolerance-related Tregs, pDCs, MDSCs, and M2 cells exhibited a strong positive correlation with the severity of lower respiratory tract infections. During the acute phase of RSV infection, a shift towards a Th2-type response, accompanied by the suppression of IFN-γ antiviral immunity, underlies airway hyperresponsiveness in a subset of susceptible infants and young children [27]. Activated Th2 cells have been observed to secrete large quantities of IL-4, IL-5, and IL-13, which in turn chemotactically attract and activate neutrophils, mast cells, basophils, and eosinophils. These cells have been shown to induce B cell antibody class switching, resulting in the secretion of substantial amounts of IgE antibodies, thereby triggering type I hypersensitivity reactions [4,28]. Our analysis results also showed a positive correlation between the severity of RSV infection and the counts of neutrophils, macrophages, mast cells, and dendritic cells. Thus, we hypothesize that the younger the infant, especially preterm infants, the more severe this immune tolerance becomes, the more prone the innate immune system is to activation, and the more inclined the cytokine secretion of the entire immune system is to Th2 polarization. This Th2 polarization tendency may facilitate the development of tolerance to self-antigens and other foreign antigens in the body, but it may also increase susceptibility to viral infections [29]. When infected with RSV, these immune system characteristics are enhanced, resulting in more severe early-onset respiratory disease (ERD) in younger infants. The results of the correlation analysis demonstrated a positive correlation between age and the severity of RSV infection, as well as between age and the number of neutrophils and mast cells. However, low correlation was observed between eosinophil levels and the severity of infection. The reason why RSV infection is more severe in younger infants is attributed to environmental exposure—particularly RSV infection itself—which may induce airway remodeling in infancy and impair the function of the developing immune system [25]. In addition to direct virus–host interactions, certain bacterial members of the respiratory microbiota may modulate the host’s response to RSV, thereby regulating inflammation and potentially influencing disease severity [30]. We have reason to believe that the severe ERD in infants and young children caused by RSV infection is the result of multiple factors. However, the immature development of the immune system is undoubtedly an extremely important contributing factor.

A total of 47 genes associated with immune cells were identified through immunological infiltration analysis. These genes were used to determine the correlation between samples and immune cells through unsupervised clustering analysis, which resulted in the population being divided into three categories. Clusters A and B were compared with cluster C, which was found to have a younger age demographic and a higher proportion of subjects in the severe group. Furthermore, a heat map displaying the expression levels of 47 immune-related genes revealed significant differences among clusters A, B and C. Given that the proportion of individuals manifesting severe and moderate symptoms in cluster B is the least substantial, the volcano plot (Figure 4F) demonstrates that, in comparison with the expression difference of cluster A/cluster C, the number and intensity of differentially expressed genes in cluster B/cluster C are considerably more pronounced. GO analysis of differentially expressed genes shows that, in comparison with cluster A/cluster B, cluster B/cluster C exhibits increased intensity of granulocyte degranulation (Supplementary-Figure S2). Consistent with previous reports, acute infection with respiratory syncytial virus (RSV) can induce degranulation of mast cells, basophils, and eosinophils. This process results in the release of a significant quantity of intracellular active mediators into surrounding tissues, leading to immune damage [4]. The expression matrices of healthy and infected individuals were used for WGCNA co-expression analysis. The purpose of this step was to screen out co-expression modules related to multiple clinical information through co-expression analysis and take the intersection with the 87 differentially expressed genes screened to screen out 61 genes.

In the context of severe symptoms of RSV infection as a quasi-time continuous index, the Mfuzz and TCseq algorithms are employed to undertake time series analysis of the expression matrix, with the objective of evaluating the two indicators of severe symptoms of RSV infection and age. The intersection of the genes obtained from the two time-series analyses was performed to obtain 25 candidate genes. The application of ROC calculation to the screening of ten genes (MCEMP1, FCGR1B, ANXA3, FAM20A, CYSTM1, GYG1, ARG1, SLPI, BMX and SMPDL3A) enabled the distinction of the severe symptoms of RSV infection. The construction of seven machine-learning algorithm models was achieved using ten genes that have been demonstrated to be capable of predicting severe symptoms of RSV infection. The prediction models were verified using three datasets: a portion of GSE246622, GSE105450 and GSE188427. The finding demonstrate that the developed model exhibits a certain predictive capacity for severe symptoms of RSV infection; however, multiple AUC values failed to exceed 0.7 due to disparities in genetic backgrounds, sampling time points, and diagnostic criteria for severe symptoms of RSV infection among the validation datasets GSE105450 and GSE188427, indicating that the predictive model still has inherent limitations that warrant further improvement.

### 4.1. MCEMP1 and CYSTM1

Mast Cell Expressed Membrane Protein 1 (MCEMP1) is a single-channel transmembrane protein involved in regulating the differentiation activities and immune responses of mast cells. Mast cells aggravate sepsis by interfering with the phagocytic activity of resident macrophages and increasing the release of inflammatory cytokines [31]. Cystinosin 1 (CYSTM1) is a novel cysteine-rich transmembrane module that plays a role in stress tolerance across eukaryotes and is significantly associated with a wide variety of immune cell types [32,33].

### 4.2. FCGR1B

Homo sapiens Fc fragment of IgG receptor 1 B (FCGR1B) is highly expressed in neutrophils. Tuberculosis promotes phagocytosis and induces severe inflammatory responses and pathological damage [34]. Neutrophils are the most abundant cell type in the airways of children [25], and it can be speculated that FCGR1B causes severe pathological damage by activating neutrophils to release a large number of inflammatory factors.

### 4.3. ANXA3 and GYG1

The protein encoded by Annexin A3 (ANXA3) is called lipocalin 3. It is a member of the calcium-binding protein family and contributes to inflammation-induced lung injury by activating nuclear factor-κB (NF-κB) [31]. In a transcriptomic study of neutrophils in peripheral blood, it was found that the expression of ANXA3 significantly increased throughout the course of sepsis [31]. Glycogen synthase 1 (GYG1) belongs to the glycogenin family and is primarily responsible for initiating glycogen synthesis. In addition, ANXA3, GYG1 and Arginase 1 (ARG1) were predicted to participate in neutrophil degranulation [35].

### 4.4. FAM20A and ARG1

There is a correlation between Family with Sequence Similarity 20 Member A (FAM20A) and ARG1, and an increase in neutrophil abundance [36,37]. In humans, ARG1 is mainly released by the liver and neutrophils. ARG catalyzes the degradation of arginine into ornithine and urea. By depleting arginine in the extracellular environment, it downregulates the expression of the CD3ζ chain in T lymphocytes, thereby inhibiting the activation and proliferation of T cells through the CD3/T-cell receptor (TCR) complex, thus inhibiting T cell activation and producing strong immunosuppression [38]. The expression of ARG1 is mainly induced by type 2 cytokines (IL-4, IL-13) and immunosuppressive cytokines (TGF-β, IL-10) [39].

### 4.5. SLPI

Secretory Leukocyte Protease Inhibitor (SLPI) is mainly expressed in the lungs, cervical mucosa, body fluids, and the skin. LPS, IL-1, TNF-α, Neutrophil elastase (NE) and neutrophil α-defensin can increase protein expression levels. Its main function is to act as a serine protease inhibitor, which can protect tissues from degradation by a variety of proteases such as cathepsin G, elastase, trypsin, chymotrypsin, chymase, and tryptase. Among them, NE, which is mainly produced by neutrophils, is regarded as the main protease target of SLPI [40]. In monocytes, SLPI can prevent the activation of NF-κB by inhibiting the degradation of NF-κB (IκB-α and IκB-β), thereby restricting the release of inflammatory factors [40].

### 4.6. BMX

Bone marrow tyrosine kinase on chromosome X (BMX) is a member of the TEC family of non-receptor tyrosine kinases [41]. In progenitor cell populations in the bone marrow and mature hematopoietic cell populations of the granulocyte/monocyte lineage, Bmx expression increases with maturation and differentiation. High levels of BMX are also found in mature peripheral neutrophils and monocytes/macrophages [42]. It regulates various cellular processes and participates in the inflammatory response cascade by regulating Toll-like receptor-induced interleukin (IL)-6 production [41,43]. Neutrophils are the most abundant cell type in the airways of children with bronchiolitis caused by respiratory syncytial virus infection; however, their exact role in the pathological response remains unclear [25].

### 4.7. SMPDL3A

Sphingomyelin phosphodiesterase acid-like 3A (SMPDL3A), a member of the acid sphingomyelinase (aSMase) family, is strongly regulated by cholesterol loading [44]. Cholesterol-activated LXR upregulates SMPDL3A expression and then selectively hydrolyzes 2′,3′-cGMP to inhibit type I interferon and NF-κB signaling pathways, thus achieving the effect of inhibiting inflammation [44,45].

Since neutrophils, macrophages and mast cells are positively correlated with the age and severe symptoms of RSV infection in infants and young children, it is reasonable to use the seven neutrophil-related genes FCGR1B, ANXA3, GYG1, FAM20A, ARG1, SLPI, and BMX as markers to predict the severe symptoms of RSV infection in infants and young children. In addition, MCEMP1, CYSTM1 and SMPDL3A also showed a high correlation with inflammation-related macrophages, mast cells and NF-κB signaling pathways, which are logically selected for prediction.

## 5. Conclusions

In conclusion, analysis of the whole blood cell transcriptome dataset GSE246622 of infantile RSV infection showed that there was a high correlation between the severe symptoms of RSV infection and the age of the infected. Immune infiltration has been demonstrated to be a marker of severity, with younger patients exhibiting more severe symptoms. This phenomenon is believed to be associated with the immune tolerance state of adaptive immunity and the over-activation of innate immunity. It is noteworthy that, although eosinophils have been documented as being present in abundance in the lungs during RSV-induced high reactivity in the lower respiratory tract, there is low correlation with severe symptoms of RSV infection. Following a thorough analysis, seven machine learning algorithm models were constructed utilizing the WGCNA, Mfuzz, TCseq, and machine learning algorithms. In the future, the scope of the study population (individuals with similar genetic backgrounds) will be further refined and restricted, the classification criteria for infection severity standardized, and the sampling process optimized to enhance the predictive performance of the models. Finally, the seven predictive models will be integrated to further improve prediction accuracy, thereby facilitating their translation into clinical applications.

## Data Availability

All human transcriptome data used for analysis in this study were obtained from the Gene Expression Omnibus (GEO) database of the National Center for Biotechnology Information (NCBI), which are publicly available de-identified datasets. The specific access information is as follows: GSE246622 (https://www.ncbi.nlm.nih.gov/geo/query/acc.cgi?acc=GSE246622, accessed on 22 August 2025), GSE188427 (https://www.ncbi.nlm.nih.gov/geo/query/acc.cgi?acc=GSE188427, accessed on 22 August 2025) and GSE105450: (https://www.ncbi.nlm.nih.gov/geo/query/acc.cgi?acc=GSE105450, accessed on 22 August 2025). All analysis codes, processed data matrices, and generated research images have been deposited in the public database Zenodoh (https://doi.org/10.5281/zenodo.16935359).

## Supplementary Materials

The following supporting information can be downloaded at https://www.mdpi.com/article/10.3390/tropicalmed11010010/s1. Supplementary-Figure S1: Correlations between IRGs and immune cells calculated by 5 algorithms, and immune cell comparison of three age group; Supplementary-Figure S2: GO analysis was used to enrich the DEGs in each pairwise comparison of Cluster A/Cluster C, Cluster A/Cluster B and Cluster B/Cluster C.
