# Supplementary material for: Analysis of the Specific Expression Profile of Immune Cells in Infants and Young Children Infected with RSV and Construction of a Disease Prediction Model
Source: Trop Med Infect Dis. 2025 Dec 29;11(1):10. doi: 10.3390/tropicalmed11010010 (PMC12846271; doi:10.3390/tropicalmed11010010)
Supplement: Supplementary file 1 [file tropicalmed-11-00010-s001.zip › tropicalmed-3851772-supplementary.pdf]

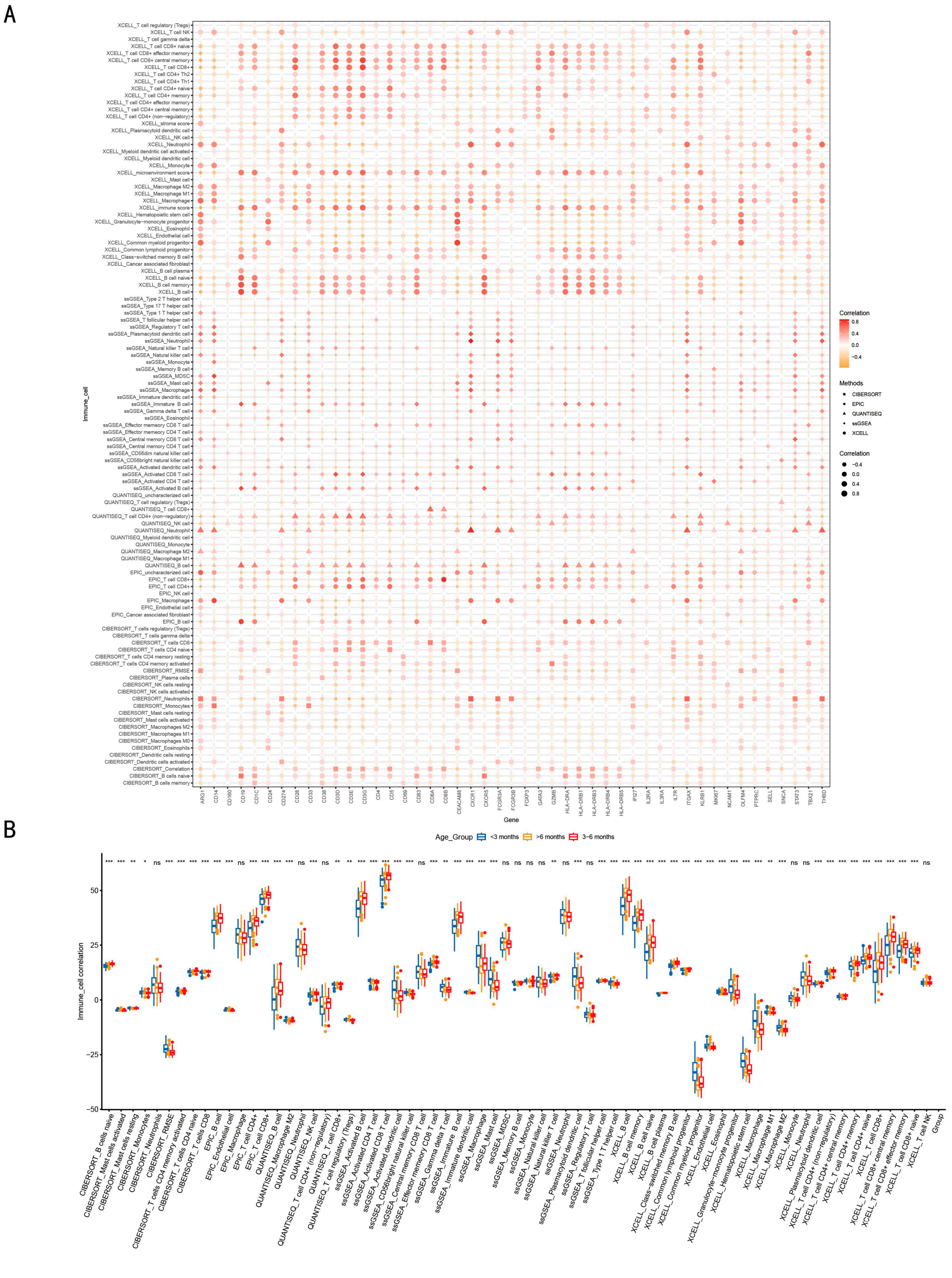

Supplementary-FigureS1 Correlations between IRGs and immune cells calculated by 5 algorithms, and immune cell comparison of three age group.

The correlations between 47 immune-related genes and multiple immune cells were analyzed using five methods: CIBERSORT, EPIC, QUANTISEQ, SSGSEA, and XCELL (A). Correlation between the expression differences of 47 immune-related genes in the four populations and their correlation with different immune cells (B).

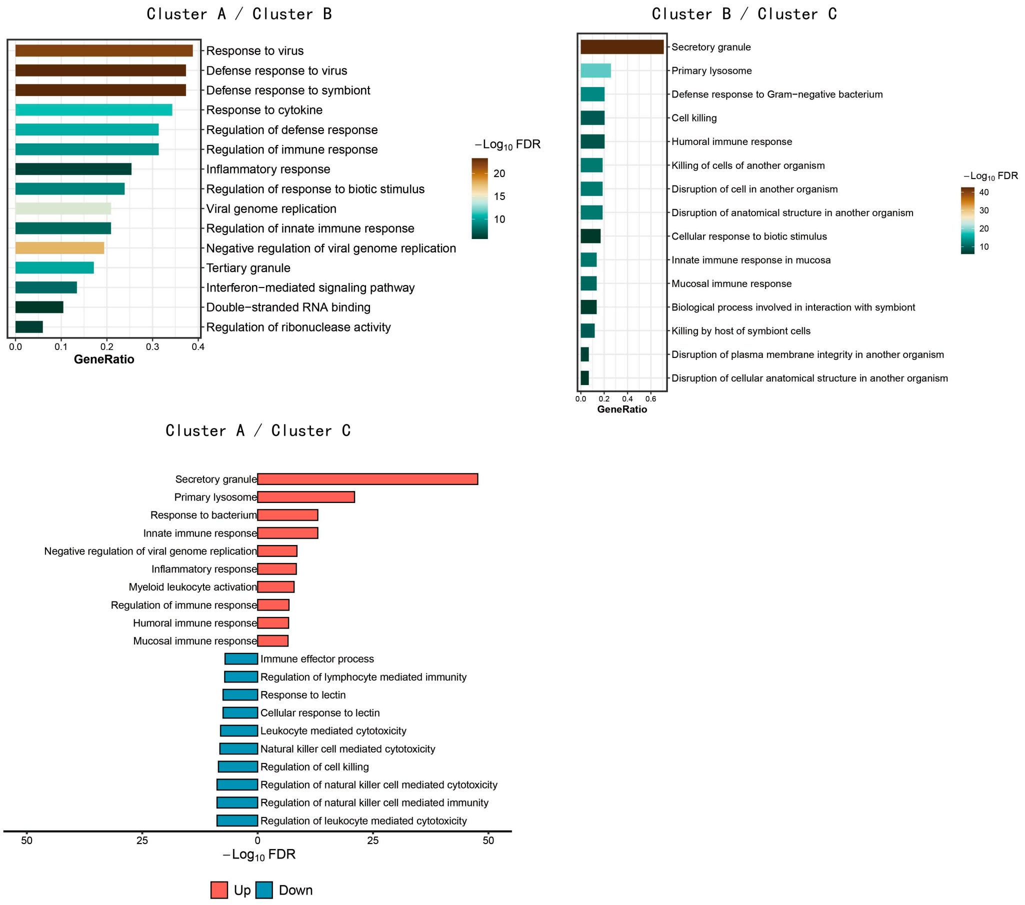

Supplementary-FigureS2 GO analysis was used to enrich the DEGs in each pairwise comparison of Cluster A/Cluster C, Cluster A/Cluster B and Cluster/Cluster C.

The three clusters classified by IRGs expression through unsupervised clustering were used to identified DEGs between every two clusters. A total of 3 panels of DEGs (Cluster A/Cluster C, Cluster A/Cluster B and Cluster/Cluster C) were employed to perform GO analysis.
